# Patterns and predictors of changes in active commuting over 12 months

**DOI:** 10.1016/j.ypmed.2013.07.020

**Published:** 2013-12

**Authors:** Jenna Panter, Simon Griffin, Alice M. Dalton, David Ogilvie

**Affiliations:** a Medical Research Council Epidemiology Unit & UKCRC Centre for Diet and Activity Research (CEDAR), Institute of Public Health, University of Cambridge, UK; b Norwich Medical School, University of East Anglia, Norwich, UK & UKCRC Centre for Diet and Activity Research (CEDAR), Institute of Public Health, University of Cambridge, UK

**Keywords:** Health promotion, Walking, Cycling, Longitudinal study, Health promotion, Behavioural change, Physical activity and health, Walking, Epidemiology, Environment design, Adults, Follow-up studies

## Abstract

**Objective:**

To assess the predictors of uptake and maintenance of walking and cycling, and of switching to the car as the usual mode of travel, for commuting.

**Methods:**

655 commuters in Cambridge, UK reported all commuting trips using a seven-day recall instrument in 2009 and 2010. Individual and household characteristics, psychological measures relating to car use and environmental conditions on the route to work were self-reported in 2009. Objective environmental characteristics were assessed using Geographical Information Systems. Associations between uptake and maintenance of commuting behaviours and potential predictors were modelled using multivariable logistic regression.

**Results:**

Mean within-participant changes in commuting were relatively small (walking: + 3.0 min/week, s.d. = 66.7; cycling: − 5.3 min/week, s.d. = 74.7). Self-reported and objectively-assessed convenience of public transport predicted uptake of walking and cycling respectively, while convenient cycle routes predicted uptake of cycling and a pleasant route predicted maintenance of walking. A lack of free workplace parking predicted uptake of walking and alternatives to the car. Less favourable attitudes towards car use predicted continued use of alternatives to the car.

**Conclusions:**

Improving the convenience of walking, cycling and public transport and limiting the availability of workplace car parking may promote uptake and maintenance of active commuting.

## Highlights

• Few studies have examined predictors of uptake and maintenance of active commuting.
• Convenient public transport and lack of parking at work predicted uptake of walking.
• Convenient cycle routes predicted uptake of cycling.
• Having a pleasant route for walking predicted maintenance of walking.
• Interventions to address these factors may help promote active commuting.

## Introduction

Everyday physical activity is important for health (Das and Horton, 2012). Active commuting (walking and cycling to work) is specifically associated with reduced morbidity and mortality (Hamer and Chida, 2008), and cross-sectional studies have shown that those who walk or cycle to work – either alone, or in combination with the car – or who commute by public transport are more physically active than those who use only the car (Pratt et al., 2012). Promoting a shift away from car use in general, and towards walking and cycling for transport in particular, therefore has potential as a public health strategy and merits further research (Das and Horton, 2012) — not least because systematic reviews of interventions have found limited evidence of effectiveness (McCormack and Shiell, 2011; Ogilvie et al., 2004, 2007; Yang et al., 2010).

Using the ecological model as a framework (Sallis and Owen, 2002), reviews of predominantly cross-sectional studies have highlighted the potential importance of a range of individual, social, and environmental factors for walking and cycling (Bauman et al., 2012; Heinen et al., 2009; Panter and Jones, 2010; Saelens and Handy, 2008). To inform the development and targeting of more effective interventions, we need to quantify changes in walking and cycling and understand the relative importance of different factors in predicting those changes, but our knowledge of these is limited (NICE, 2012; Shephard, 2008). Perceptions of the neighbourhood environment were associated with uptake and maintenance of walking for transport (Cleland et al., 2008), while proximity to facilities for physical activity was associated with more favourable trends in walking in older adults (Li et al., 2005; Michael et al., 2010). Studies of people relocating to new residential environments found that those moving to areas with higher street connectivity reported more walking,(Wells and Yang, 2008), while those moving to areas with higher residential density, street connectivity and park access were more likely to take up cycling (Beenackers et al., 2012).

These few previous studies are limited by small sample sizes (Wells and Yang, 2008) or a focus on specific population groups (Cleland et al., 2008; Li et al., 2005; Michael et al., 2010) or behaviours (Beenackers et al., 2012). Using data from the *Commuting and Health in Cambridge* study, we aimed to describe changes in walking and cycling to and from work in a cohort of commuters and assess the predictors of uptake and maintenance of walking, cycling and use of alternatives to the car for commuting.

## Methods

### Study setting, participant recruitment and data collection

Cambridge has a distinct cycling culture related to its flat topography and large university population. The *Commuting and Health in Cambridge* study protocol, recruitment and data collection procedures and baseline results have been reported elsewhere (Ogilvie et al., 2010; Panter et al., 2011; Yang et al., 2012). Briefly, adults aged 16 and over who lived within 30 km of the city centre and travelled to work in Cambridge were recruited, predominantly through workplaces, and received postal questionnaires between May and October 2009 (t_1_) and again one year later (t_2_). Individual data collection was matched to the same week of the year wherever possible to minimise any seasonal differences in behaviour. To avoid breaching data protection legislation and to assure participants of the study's independence, commuters were not recruited using employer-based sampling frames such as staff databases but were invited to opt in to the study through a variety of strategies including recruitment stands, advertisements and emails distributed through corporate mailing lists. A variety of workplaces contributed to participant recruitment. These included local authorities, healthcare providers, retail outlets and institutions of higher and further education distributed across a range of city centre and urban fringe locations in Cambridge. Of the 2163 people who registered their interest in taking part in the study, 1582 met the inclusion criteria and were sent a questionnaire at t_1_; of these, 1164 (74%) provided consent and returned a completed baseline questionnaire.

### Outcomes: uptake and maintenance of walking, cycling and use of alternatives to the car

At both time points participants were asked to report the travel modes used on each commuting journey over the last seven days. If participants walked or cycled for any part of their journeys they reported the average time spent doing so per trip, from which total weekly times spent walking and cycling at t_1_ and t_2_ and change scores (t_2 −_t_1_) were computed. Change scores of > ± 300 min/week (n = 9) were truncated to 300. The most frequently reported travel mode or combination of modes (hereafter referred to as ‘usual’ mode(s)) used at each time point was also computed (Appendix A). Six binary outcome measures – uptake and maintenance of walking and of cycling (based on time) and of use of alternatives to the car (based on usual mode) – were subsequently derived (Table 1).

### Predictors

#### Overview

Potential predictors were measured at baseline and chosen because they represented constructs within the socio-ecological model (Sallis and Owen, 2002) and had support in the literature (Heinen et al., 2009; Panter and Jones, 2010; Saelens and Handy, 2008).

#### Individual and household characteristics

Date of birth, gender, highest educational qualification, housing tenure, household composition, access to cars and bicycles, possession of a driving licence and self-reported height and weight were assessed by questionnaire. Age and body mass index (BMI) (kg/m^2^) were calculated and participants were assigned to one of three categories of weight status (World Health Organisation, 2000).

#### Psychological measures relating to car use

Using a five-point Likert scale, participants reported their agreement with eight statements on using the car for the commute next time (for example: ‘It would be good to use the car’) representing four constructs (perceived behavioural control, intention, attitude and subjective norms; two items per construct) from the theory of planned behaviour (Hardeman et al., 2009). Habit strength for car commuting was summarised using a binary variable derived from participants' agreement on the same scale with seven statements derived from the habit strength index (Panter et al., 2013; Verplanken and Orbell, 2003).

#### Perceptions of the environment

Using a five-point Likert scale, participants reported their level of agreement with seven statements describing the environment along their commuting route (for example: ‘There is little traffic’). Responses to positively worded items were collapsed such that those who ‘strongly agreed’ or ‘agreed’ with an item were compared to those who ‘strongly disagreed’, ‘disagreed’ or ‘neither disagreed or agreed’, and vice versa for negatively worded items. Participants also reported the car parking provision at their workplace (free, paid or no parking) and the distance between their home and workplace, summarised as a categorical measure (< 5 km, 5–20 km and > 20 km) to distinguish relatively long or short trips (Panter et al., 2013).

#### Objectively assessed measures of the environment

Using a geographical information system (ArcGIS, version 9.3), characteristics of the areas surrounding the home, workplace and route to work were derived using t_1_ postcodes (Appendix B). Variables were included if they were associated with travel behaviour in cross-sectional analyses of the baseline sample: those relating to the home location (urban–rural status, area-level deprivation, road junction density, distance to the nearest railway station and the nearest bus stop, and frequency of bus services), the workplace location (density of destinations within walking distance) and the geographical context of the commuting route (Dalton et al., 2013; Panter et al., 2011).

### Analysis

All analyses were conducted in Stata 11.1. Differences in baseline characteristics between participants with and without follow-up data were tested using *t* tests, *χ*^2^ tests or Mann–Whitney U tests. One-way analysis of variance was used to test for differences between change in usual mode(s) and in time spent walking or cycling.

Associations between potential predictors and all outcomes were assessed using logistic regression models, initially adjusted for age and sex. Route characteristics were matched to the behaviour of interest; thus walking models included pleasantness and convenience of routes for walking and convenience of public transport, while cycling models included convenience of routes for cycling. All variables significantly associated at *p* < 0.25 (in the case of categorical variables, p < 0.25 for heterogeneity between groups) (Hosmer and Lemeshow, 1989) were carried forward into multivariable regression models. No adjustment was made for clustering by workplace, as preliminary multilevel models suggested no evidence of this.

Relocation can alter the length of a commute or the route taken. As a sensitivity analysis, we identified participants who reported different home or work postcodes at t_1_ and t_2_ corresponding to different locations. Excluding these movers (n = 155) from analysis made no substantial difference to the direction or size of associations, hence the results presented include these participants.

## Results

### Sample characteristics

Of the 1164 participants who returned questionnaires at t_1_, 704 (60.5%) completed questionnaires at t_2_ and 655 provided information on commuting at both t_1_ and t_2_ and were included in this analysis (Table 2). Those included were more likely to be older (mean age of 43.6 years versus 40.5 years, *p* = 0.01) and to own their own home (75.1% versus 71.8%, *p* = 0.01) than those who did not participate at t_2_. There were no significant differences in gender, educational qualifications, weight status, car ownership or time spent walking or cycling at baseline.

### Changes in weekly time spent walking and cycling and usual commuting mode(s)

**Fig. 1:**
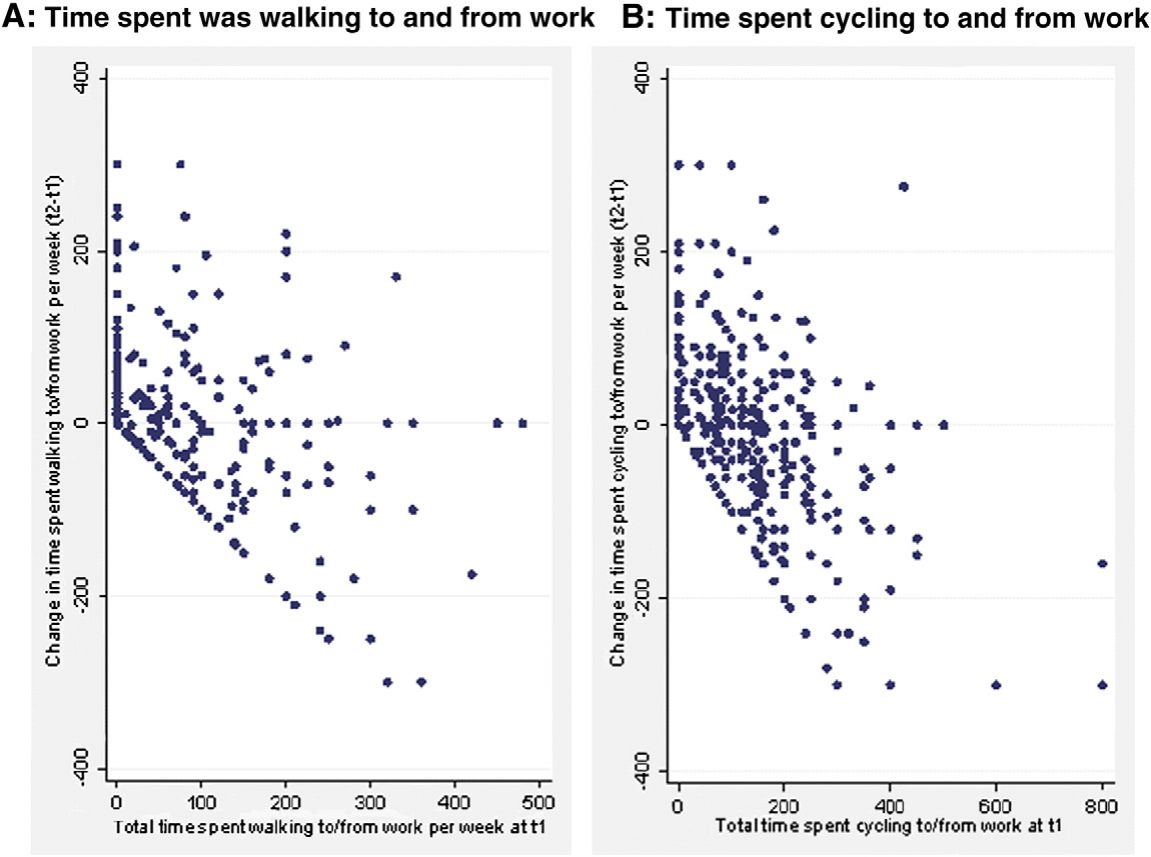
Scatterplot of change spent in time against time reported at baseline for A) walking and B) cycling on the commute.

Changes in time spent walking and cycling were symmetrically distributed. Many participants had change values of 0 min/week, reflecting either: (i) no walking (or cycling) at t_1_ and t_2_ or (ii) exactly the same number of trips and average duration of walking (or cycling) per trip at t_1_ and t_2_. Mean change values were relatively small (walking: + 3.0 min/week, s.d. = 66.7, *p* = 0.24; cycling: − 5.3 min/week, s.d. = 74.7, *p* = 0.07). Those who reported more time walking or cycling on the journey to work at t_1_ tended to report less at t_2_ (Fig. 1). Generally, changes reflected a combination of changes in trip frequency and average duration per trip, although many cyclists reported the same number of trips but different durations (Appendix C).

**Fig. 2:**
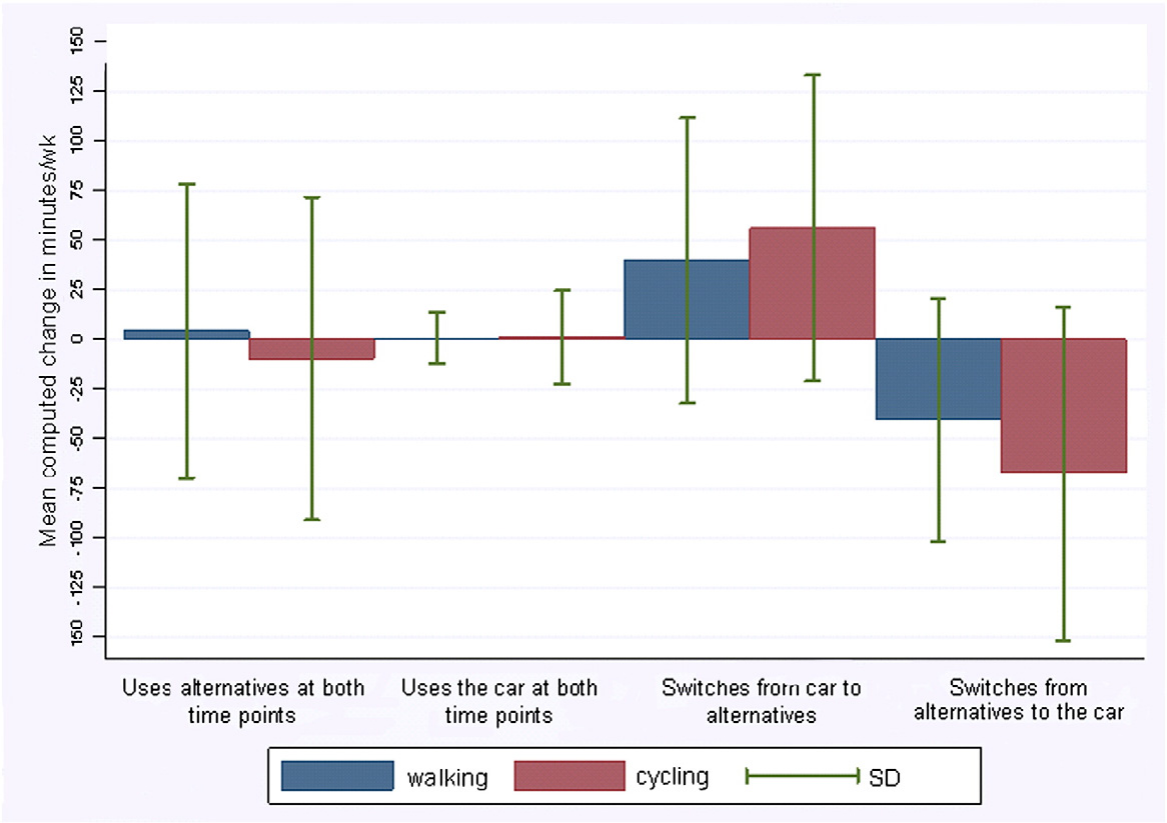
Mean changes in computed time spent walking and cycling according to modal shift category.

Most participants reported the same usual mode at t_1_ and t_2_. 21% and 68% used the car and alternatives to the car at both t_1_ and t_2_ respectively, whilst 6% switched to the car at t_2_ and 6% switched away from the car. Changes in time spent walking and cycling differed according to change in usual mode (*p* < 0.001 for both walking and cycling; Fig. 2). Those who switched away from the car reported substantial mean increases in walking and cycling, whereas those switching to the car reported substantial mean decreases.

### Predictors of uptake and maintenance of walking, cycling and use of alternatives to the car

Results for uptake and maintenance of walking, cycling and use of alternatives to the car are presented in Tables 3, 4 and 5 respectively. Commuters with no children in the household or who reported convenient public transport or a lack of free workplace parking were more likely to take up walking. Those reporting convenient cycle routes or living in areas objectively assessed to have more frequent bus services were more likely to take up cycling. Older participants, those with a degree, and those who reported convenient cycle routes or a lack of free workplace parking were more likely to take up alternatives to the car.

In general, only a few of the potential predictors were associated with maintenance of more active travel behaviours. Only those who reported that it was pleasant to walk on the route to work were significantly more likely to maintain walking, whereas none of the potential predictors were associated with maintenance of cycling. Area-level deprivation and less favourable attitudes towards car use predicted continued use of alternatives to the car.

## Discussion

### Principal findings

Small average changes in weekly time spent walking or cycling on the commute were observed over the 12-month period. However, among participants who switched from the car to an alternative as their usual mode of transport, the mean increases in active travel time were substantial and of a similar order of magnitude as the effect sizes reported in controlled studies of interventions to promote walking for transport (15–30 min/week) (Ogilvie et al., 2007). Sociodemographic factors predicted uptake and maintenance of use of alternatives to the car, and having no children in the household predicted uptake of walking. Supportive transport environments predicted uptake of walking and cycling. Lack of free workplace parking predicted uptake of walking and of alternatives to the car. Less favourable attitudes towards car use predicted maintenance of using alternatives to the car.

### Quantifying change in walking and cycling

We cannot be certain to what extent the computed changes in travel time represent true changes or the effects of measurement error. Although there are no validated measures of transport-specific physical activity behaviours, the fact that few participants reported small non-zero changes (± 15 min/week) suggests that commuters' estimates of such a frequently-performed and relatively habitual behaviour may well have been relatively accurate.

Modest increases in individuals' daily walking or cycling time could have important public health implications when aggregated at a population level (Rose, 1992). They may also be important for individual health outcomes, although more rigorous longitudinal evidence is required to assess whether increases in active commuting result in increases in overall physical activity and health at an individual level (Shephard, 2008).

### Potential targets for intervention

Previous reviews of the environmental correlates of walking and cycling have generally reported inconsistent or null associations (Heinen et al., 2009; Panter and Jones, 2010; Saelens and Handy, 2008). In keeping with the findings of one more recent review, however (McCormack and Shiell, 2011), our longitudinal findings suggest several plausible targets for environmental interventions, such as restricting workplace parking and providing convenient routes for cycling, convenient public transport and pleasant routes for walking (Ogilvie et al., 2007; Yang et al., 2010). Their effects on commuting behaviour and physical activity are largely unknown and should be assessed in future studies.

We also found that commuters with less favourable attitudes towards car use were more likely to continue using alternatives to the car, possibly due to perceived lack of choice. Changing attitudes may be difficult, however, particularly in the car-orientated environments that typify many developed countries. The provision of more supportive environments for walking and cycling may itself result in changes in attitudes or perceptions over time and this seems an important avenue for future research. While a combination of observational analyses of longitudinal data of this kind may strengthen the evidence base for a causal pathway linking environmental change to behaviour change, further research should also elucidate the mediating mechanisms in quasi-experimental studies of actual interventions.

Other characteristics were also important predictors of behaviour. Those who lived in more deprived areas were more likely to continue using alternatives to the car, while older adults and those without children were more likely than those with children to take up walking to work. Qualitative research in this sample and elsewhere (Cleland et al., 2008; Guell et al., 2012; Pooley et al., 2012) has highlighted the importance of the social context in shaping travel behaviour. The tailoring and evaluation of interventions to promote walking and cycling should take account of these contextual considerations.

### Strengths and limitations

This is one of the few longitudinal studies to provide a detailed quantification of changes in active commuting or to assess the predictors of uptake and maintenance of walking, cycling and use of alternatives to the car on the commute. Our use of a range of self-reported and objectively measured potential predictors specific to commuting, in a large cohort of healthy working commuters from urban and rural areas is an important strength. We also classified change using two complementary metrics: a detailed continuous measure of time spent walking or cycling; and a categorical measure based on the usual mode of travel, that might more accurately reflect habitual travel behaviour.

Our findings may not be generalisable to other contexts where cycling is less prevalent. Only 56% of participants provided data at follow-up, and although travel mode was not associated with dropout, the attrition of the cohort limits the generalisability of our observations. Our sample also contained a higher proportion of participants educated to degree level and a smaller proportion of obese adults than the population of Cambridgeshire (Office of National Statistics, 2011). While our measure of time spent walking and cycling improves on many instruments used previously (Ogilvie et al., 2004), we did not collect information on the time spent walking or cycling on each day. We also lacked information on measures of socio-economic status or workplace facilities for cyclists, which may influence commuting behaviour. Relatively few participants had changed their usual travel mode(s), which may have limited our power to detect associations. Further investigation in larger samples with data collected at multiple time points over a longer time period would be warranted.

## Conclusions

In this longitudinal study, we found a lack of empirical support for many of the putative predictors of travel behaviour change suggested by findings from cross-sectional studies. Only a few were found to be important; based on these findings, interventions to restrict workplace parking and provide convenient routes for cycling, convenient public transport and pleasant routes for walking to work appear to hold promise. Their effects on travel behaviour are, however, largely unknown and further studies are required to establish these.

## Conflict of interest statement

The authors declare that there are no conflicts of interest.

## Figures and Tables

**Table 1:** Details of outcome measures used.

Outcome	Variable used to define change	Predictor group		Reference group		Sample size used in analysis^a^
Description	Sample size^b^	Description	Sample size^b^
Uptake of walking	Weekly time spent walking	Increased walking (from 0 at t_1_ to > 0 at t_2_) (‘took up walking’)	72	Spent no time walking at either time point (‘no walking’)	401	470
Uptake of cycling	Weekly time spent cycling	Increased cycling (from 0 at t_1_ to > 0 at t_2_) (‘took up cycling’)	33	Spent no time cycling at either time point (‘no cycling’)	268	293
Uptake of alternatives to the car	Most frequently reported mode(s)	Shifted from car to alternative usual mode	37	Car user at both time points	137	174
Maintenance of walking	Weekly time spent walking	Reported same time walking at both time points, where time > 0 OR increased walking, where time > 0 at t_1_ (‘maintained their walking’)	73	Decreased time spent walking (‘reduced or gave up walking’)	109	181
Maintenance of cycling	Weekly time spent cycling	Reported same time cycling at both time points, where time > 0 OR increased cycling, where time > 0 at t_1_ (‘maintained their cycling’)	186	Decreased time spent cycling (‘reduced or gave up cycling’)	168	347
Maintenance of use of alternatives to the car	Most frequently reported mode(s)	Used alternative to car at both time points	444	Switched to car as usual mode	37	462

Data collected in 2009 and 2010 in Cambridge, UK.

a Sample size refers to actual number of participants used in maximally adjusted models (those with complete data for all predictors included in the model).

b Sample size refers to potential numbers of participants in each group (not accounting for missing data in potential predictors).

**Table 2:** Characteristics of participants with data at both time points.

	Percentage (n)
*Individual characteristics*	
Gender (n = 655)	
Male	31.6 (207)
Female	68.4 (448)
Mean age (s.d.)	43.65 (11.3)
Highest educational qualification (n = 655)	
Less than degree	26.3 (172)
Degree or higher	73.7 (483)
Weight status (n = 655)	
Normal or underweight	63.3 (415)
Overweight or obese	36.7 (240)

*Household characteristics*	
Number of children in household (n = 655)	
None	72.0 (472)
One or more	28.0 (183)
Home ownership (n = 655)	
Does not own home	24.9 (163)
Home owner	75.1 (492)
Number of cars in household (n = 655)	
None	14.8 (97)
One car or more	84.2 (558)
Home location (n = 655)	
Urban	64.7 (424)
Rural	35.3 (231)
Mean (s.d.) self-reported distance between home and work (km)	13.1 (11.3)

*Walking and cycling*	
Change in time spent walking to and from work (n = 654; median = 0 min/week, IQR = 0,0)	
No walking reported at either time point	61.2 (401)
Exactly the same non-zero time at both time points	2.1 (14)
Increase in weekly walking time	20.0 (131)
Decrease in weekly walking time	16.7 (108)
Change in time spent cycling to and from work (n = 655; median = 0 min/week, IQR = –10,0)	
No cycling reported at either phase 1 or phase 2	41.0 (268)
Exactly the same non-zero time at both time points	9.6 (63)
Increase in weekly cycling time	23.0 (151)
Decrease in weekly cycling time	26.4 (173)

IQR: interquartile range. Data collected in 2009 and 2010 in Cambridge, UK.

**Table 3:** Uptake and maintenance of walking.

		Uptake of walking OR (95% CI)		Maintenance of walking OR (95% CI)	
Minimally adjusted +	Maximally adjusted ‡	Minimally adjusted +	Maximally adjusted ‡
*Personal and household characteristics*					
Age (years)		n/a	1.01 (0.98, 1.03)	n/a	1.00 (0.97, 1.02)
Gender	Male		1.0		1.0
Female	n/a	1.11 (0.61, 2.03)	n/a	1.55 (0.74, 3.23)
Weight status	Overweight or obese	1.0		1.0	
Normal or underweight	1.37 (0.79, 2.40)	–	1.11 (0.60, 2.06)	–
Highest educational qualification	Less than degree	1.0	1.0	1.0	
Degree or higher	0.70 (0.40, 1.22)	0.74 (0.41, 1.35)	1.12 (0.57, 2.23)	–
Number of children	One or more	1.0	1.0	1.0	1.0
None	2.20 (1.56, 4.17)	2.18 (1.08, 4.39)	1.87 (0.86, 4.09)	1.74 (0.79, 3.85)
Cars	One or more	1.0	1.0	1.0	
None	1.62 (0.80, 3.29)	1.10 (0.49, 2.46)	0.63 (0.28, 1.38)	–
Home ownership	Does not own home	1.0	1.0	1.0	
Owns home	1.67 (0.90, 3.08)	1.30 (0.66, 2.53)	1.59 (0.72, 3.51)	–

Objectively measured environment					
Home location	Rural	1.0	1.0	1.0	
Urban	1.41 (0.82, 2.46)	1.18 (0.61, 2.28)	0.94 (0.49, 1.80)	–
Area-level deprivation	More affluent	1.0		1.0	
Less affluent	0.88 (0.53, 1.47)	–	1.26 (0.69, 2.31)	–
Junction density around home	Lower	1.0	1.0	1.0	
Higher	1.51 (0.91, 2.52)	1.13 (0.63, 2.02)	1.15 (0.63, 2.09)	–
Distance to nearest railway station from home	Further	1.0		1.0	
Closer	0.99 (0.60, 1.64)	–	1.00 (0.55, 1.84)	–
Distance to nearest bus stop from home	Further	1.0		1.0	
Closer	1.11 (0.67, 1.83)	–	1.05 (0.57, 1.93)	–
Frequency of bus services around home	Less frequent	1.0		1.0	
More frequent	1.00 (0.60, 1.66)	–	0.87 (0.48, 1.58)	–
Destinations within walking distance around work	Lower density	1.0		1.0	
Higher density	1.30 (0.78, 2.15)	–	0.93 (0.51, 1.71)	–
Geographical context of commute	Commuting to the heart from within the city	1.0		1.0	
Commuting to the outskirts from within the city	0.77 (0.37, 1.59)		0.76 (0.31, 1.90)	–
Commuting to the heart from outside the city	1.43 (0.68, 3.00)	–	0.78 (0.34, 1.78)	–
Commuting to the outskirts from outside the city	0.78 (0.38, 1.62)		1.10 (0.49, 2.44)	

*Self-reported measures of the environment*					
Pleasant to walk	SD/D/N	1.0		1.0	1.0
A/SA	1.06 (0.63, 1.78)	–	2.48 (0.76, 8.15)	2.34 (1.07, 5.11)
Convenient public transport	SD/D/N	1.0	1.0	1.0	
A/SA	2.46 (1.47, 4.13)	2.47 (1.44, 4.25)	0.72 (0.39, 1.31)	–
No convenient walking routes	A/SA	1.0		1.0	
SD/D/N	0.88 (0.53, 1.46)	–	1.82 (0.42, 7.86)	–
Little traffic	SD/D/N	1.0		1.0	
A/SA	0.70 (0.29, 1.71)	–	1.17 (0.63, 2.16)	–
Safe to cross the road	SD/D/N	1.0		1.0	
A/SA	1.24 (0.75, 2.07)	–	0.94 (0.51, 1.73)	–
Self-reported distance from home to work	Over 20 km	1.0		1.0	
5.0–20 km	0.45 (0.24, 0.87)	–	0.97 (0.46, 2.07)	
Under 5 km	0.72 (0.40, 1.33)	–	0.79 (0.39, 1.60)	–
Workplace car parking	Free	1.0	1.0	1.0	
None or paid-for	2.35 (1.34, 4.12)	2.04 (1.12, 3.71)	1.17 (0.58, 2.36)	–

*Psychological measures relating to car use*					
Intention score	Strong intentions	1.0		1.0	
Weak intentions	0.96 (0.57, 1.62)	–	1.35 (0.74, 2.47)	–
Attitude score	More favourable attitudes	1.0		1.0	
Less favourable attitudes	1.07 (0.64, 1.80)	–	1.08 (0.60, 1.97)	–
PBC score	Higher PBC score	1.0	1.0	1.0	
Lower PBC score	1.51 (0.90, 2.53)	0.94 (0.51, 1.73)	0.85 (0.46, 1.56)	–
Social norm score	Higher social norms	1.0		1.0	
Lower social norms	1.17 (0.69, 1.98)	–	0.72 (0.40, 1.33)	–
Habit strength	Higher habit strength	1.0		1.0	
Lower habit strength	0.97 (0.58, 1.63)	–	1.14 (0.62, 2.07)	–

PBC: perceived behavioural control; +: adjusted for age and sex only; ‡: adjusted for all other variables included in the model; SA: strongly agree; A: agree; N: neither; SD: strongly disagree; D: disagree. –: not significant in minimally adjusted models; n/a: models adjusted only for age and sex not presented. Data collected in 2009 and 2010 in Cambridge, UK.

**Table 4:** Uptake and maintenance of cycling.

		Uptake of cycling OR (95% CI)		Maintenance of cycling OR (95% CI)	
Minimally adjusted +	Maximally adjusted ‡	Minimally adjusted +	Maximally adjusted ‡
*Personal and household characteristics*					
Age (years)		n/a	1.00 (0.96, 1.04)	n/a	0.99 (0.97, 1.01)
Gender	Male		1.0		1.0
Female	n/a	1.38 (0.51, 3.74)	n/a	1.21 (0.77, 1.88)
Weight status	Overweight or obese	1.0		1.0	
Normal or underweight	0.98 (0.89, 1.08)	–	0.85 (0.60, 1.22)	–
Highest educational qualification	Less than degree	1.0	1.0	1.0	
Degree or higher	1.67 (0.71, 3.89)	1.75 (0.68, 4.51)	1.24 (0.73, 2.10)	–
Number of children	One or more	1.0		1.0	
None	0.77 (0.34, 1.71)	–	1.01 (0.63, 1.59)	–
Cars	One or more	1.0	1.0	1.0	
None	2.06 (0.80, 5.30)	0.50 (0.13, 2.00)	1.05 (0.60, 1.86)	–
Home ownership	Does not own	1.0	1.0	1.0	
Owns home	3.04 (1.34, 6.94)	2.32 (0.87, 6.19)	0.95 (0.54, 1.68)	–

*Objectively measured environment*					
Home location	Rural	1.0		1.0	
Urban	1.44 (0.68, 3.05)	–	1.15 (0.70, 1.91)	–
Area-level deprivation	More affluent	1.0		1.0	
Less affluent	1.04 (0.50, 2.17)	–	1.20 (0.78, 1.85)	–
Junction density around home	Lower	1.0		1.0	
Higher	1.03 (0.50, 2.15)	–	0.86 (0.56, 1.31)	–
Distance to nearest railway station from home	Further	1.0		1.0	
Closer	1.64 (0.79, 3.41)	0.94 (0.35, 2.55)	0.99 (0.65, 1.53)	–
Distance to nearest bus stop from home	Further	1.0		1.0	
Closer	0.3 (0.45, 1.94)	–	1.06 (0.70, 1.63)	
Frequency of bus services around home	Less frequent	1.0	1.0	1.0	–
More frequent	3.64 (1.73, 7.67)	2.59 (0.99, 6.78)	0.91 (0.58, 1.43)	
Destinations within walking distance around work	Lower density	1.0		1.0	
Higher density	1.03 (0.49, 2.16)	–	0.88 (0.58, 1.34)	–
Geographical context of commute	Commuting to the heart from within the city	1.0	1.0	1.0	
Commuting to the outskirts from within the city	1.34 (0.42, 4.30)	1.27 (0.33, 4.85)	0.76 (0.31, 1.90)	–
Commuting to the heart from outside the city	0.36 (0.10, 1.27)	1.53 (0.23, 10.09)	0.78 (0.34, 1.78)	–
Commuting to the outskirts from outside the city	0.43 (0.15, 1.26)	1.34 (0.22, 8.10)	1.10 (0.49, 2.44)	

*Self-reported measures of the environment*					
Dangerous to cycle	SD/D/N	1.0	1.0	1.0	
A/SA	2.16 (0.88, 5.29)	1.49 (0.52, 4.22)	0.93 (0.59, 1.46)	–
Convenient cycle routes	SD/D/N	1.0	1.0	1.0	
A/SA	2.79 (1.34, 5.84)	2.48 (1.04, 5.93)	1.14 (0.71, 1.84)	–
Little traffic	A/SA	1.0		1.0	
SD/D/N	1.88 (0.38, 9.35)	–	1.12 (0.61, 2.06)	–
Safe to cross the road	SD/D/N	1.0		1.0	
A/SA	1.40 (0.67, 2.95)	–	1.14 (0.74, 1.74)	–
Self-reported distance from home to work	Over 20 km	1.0	1.0	1.0	1.0
5.0–20 km	0.96 (0.36, 2.54)	0.85 (0.29, 2.56)	1.12 (0.51, 2.48)	1.14 (0.50, 2.56)
Under 5 km	3.94 (1.67, 9.31)	2.36 (0.32, 17.60)	1.45 (0.67, 3.16)	1.57 (0.70, 3.53)
Workplace car parking	Free	1.0	1.0	1.0	1.0
None or paid-for	1.83 (0.83, 4.03)	1.91 (0.73, 4.99)	0.69 (0.44, 1.08)	0.67 (0.42, 1.05)

*Psychological measures relating to car use*					
Intention score	Strong intentions	1.0	1.0	1.0	
Weak intentions	2.29 (1.08, 4.86)	1.32 (0.27, 6.53)	1.19 (0.76, 1.87)	–
Attitude score	More favourable attitudes	1.0	1.0	1.0	
Less favourable attitudes	2.51 (1.18, 5.33)	1.32 (0.37, 4.76)	1.17 (0.74, 1.87)	–
PBC score	Higher PBC score	1.0	1.0	1.0	1.0
Lower PBC score	1.97 (0.94, 4.14)	1.26 (0.36, 4.39)	0.76 (0.49, 1.18)	0.70 (0.44, 1.10)
Social norm score	Higher social norm	1.0	1.0	1.0	
Lower social norm	2.05 (0.93, 4.53)	0.51 (0.14, 1.82)	1.06 (0.69, 1.62)	–
Habits	Higher habit strength	1.0	1.0	1.0	
Lower habit strength	2.10 (0.98, 4.51)	0.64 (0.13, 3.29)	1.10 (0.70, 1.72)	–

PBC: perceived behavioural control; +: adjusted for age and sex only; ‡: adjusted for all other variables included in the model; SA: strongly agree; A: agree; N: neither; SD: strongly disagree; D: disagree. –: not significant in minimally adjusted models; n/a: models adjusted only for age and sex not presented. Data collected in 2009 and 2010 in Cambridge, UK.

**Table 5:** Predictors of uptake and maintenance of use of alternatives to the car.

		Uptake of alternatives to the car OR (95% CI)		Maintenance of alternatives to the car OR (95% CI)	
Minimally adjusted +	Maximally adjusted^‡^	Minimally adjusted +	Maximally adjusted^⁎^
*Personal and household characteristics*					
Age (years)		n/a	1.09 (1.03, 1.15)	n/a	0.98 (0.95, 1.02)
Gender	Male		1.0		
Female	n/a	0.47 (0.15, 1.45)	n/a	0.83 (0.34, 2.03)
Weight status	Overweight or obese	1.0		1.0	
Normal or underweight	1.41 (0.66, 3.05)	–	1.48 (0.75, 2.95)	–
Highest educational qualification	Less than degree	1.0	1.0	1.0	
Degree or higher	1.83 (0.78, 4.29)	3.52 (1.01, 12.26)	1.30 (0.61, 2.75)	–
Number of children	One or more	1.0		1.0	1.0
None	1.17 (0.50, 2.71)	–	1.91 (0.94, 3.89)	0.49 (0.22, 1.12)
Home ownership	Does not own	1.0	1.0	1.0	
Owns home	4.43 (1.69, 11.63)	3.33 (0.84, 13.25)	1.53 (0.60, 3.94)	–

*Neighbourhood characteristics*					
Home location	Rural	1.0		1.0	1.0
Urban	1.44 (0.68, 3.04)	–	2.14 (1.06, 4.29)	1.42 (0.42, 4.74)
Area-level deprivation	More affluent	1.0	1.0	1.0	1.0
Less affluent	1.85 (0.87, 3.94)	1.64 (0.56, 4.85)	2.78 (1.32, 5.85)	2.49 (1.02, 6.07)
Junction density around home	Lower	1.0	–	1.0	–
Higher	1.39 (0.67, 2.89)	1.08 (0.55, 2.13)
Distance to nearest railway station from home	Further	1.0	–	1.0	1.0
Closer	1.07 (0.47, 2.42)	2.37 (1.19, 4.74)	1.28 (0.50, 3.26)
Distance to nearest bus stop from home	Further	1.0	–	1.0	1.0
Closer	0.95 (0.44, 2.02)	1.67 (0.84, 3.30)	1.86 (0.82, 4.24)
Frequency of bus services around home	Less frequent	1.0	1.0	1.0	–
More frequent	1.87 (0.84, 4.17)	1.86 (0.48, 7.11)	0.72 (0.36, 1.47)
Destinations within walking distance around work	Lower density	1.0	1.0	1.0	1.0
Higher density	1.56 (0.74, 3.27)	5.37 (0.02, 146.71)	1.56 (0.79, 3.09)	1.52 (0.27, 8.66)

*Workplace characteristics*					
Self-reported distance from home to work	Over 20 km	1.0	1.0	1.0	1.0
5.0–20 km	0.76 (0.33, 1.77)	0.60 (0.17, 2.11)	0.98 (0.43, 2.23)	0.61 (0.19, 1.99)
Under 5 km	8.88 (2.41, 32.67)	6.22 (0.38, 101.25)	2.89 (1.13, 7.41)	0.61 (0.12, 2.98)
Workplace car parking	Free	1.0	1.0	1.0	
No or paid for	4.42 (1.97, 9.95)	22.62 (4.42, 115.78)	0.81 (0.38, 1.72)	
Geographical context of commute	Commuting to the heart from within the city	1.0	1.0	1.0	1.0
Commuting to the outskirts from within the city	0.49 (0.09, 2.75)	0.86 (0.01, 532.09)	0.69 (0.24, 2.00)	1.36 (0.20, 9.17)
Commuting to the heart from outside the city	0.21 (0.04, 1.05)	1.01 (0.04, 24.82)	0.43 (0.15, 1.25)	1.37 (0.26, 7.31)
Commuting to the outskirts from outside the city	0.18 (0.04, 0.85)	0.79 (0.00, 419.06)	0.29 (0.11, 0.81)	1.52 (0.14, 16.88)

*Perceptions of route environment*					
It is pleasant to walk	SD/D/N	1.0		1.0	
SA/A	1.08 (0.49, 2.39)	–	1.37 (0.69, 2.72)	–
It is dangerous to cycle	SA/A	1.0		1.0	
SD/D/N	0.47 (0.13, 1.74)	–	1.22 (0.54, 2.77)	–
There are convenient cycle routes	SD/D/N	1.0	1.0	1.0	
SA/A	3.81 (1.70, 8.52)	4.65 (1.45, 14.92)	1.43 (0.72, 2.84)	–
There is little traffic	SD/D/N	1.0		1.0	
SA/A	1.92 (0.44, 8.42)	–	2.22 (0.52, 9.54)	–
There is convenient public transport	SD/D/N	1.0		1.0	
SA/A	1.02 (0.41, 2.54)	–	1.44 (0.71, 2.94)	–
There are no convenient routes for walking	SA/A	1.0		1.0	1.0
SD/D/N	1.60 (0.70, 3.64)	–	2.68 (1.34, 5.39)	1.73 (0.77, 3.86)
It is safe to cross the	SD/D/N	1.0	1.0	1.0	
road	SA/A	1.76 (0.82, 3.77)	0.85 (0.28, 2.63)	1.06 (0.54, 2.10)	–

*Psychological measures relating to car use*					
Intention score	Strong intentions	1.0		1.0	1.0
Weak intentions	2.41 (0.39, 14.74)	–	4.09 (1.93, 8.68)	1.58 (0.49, 5.09)
Attitude score	More favourable attitudes	1.0	1.0	1.0	1.0
Less favourable attitudes	2.98 (0.94, 9.44)	1.22 (0.17, 9.09)	5.06 (2.35, 10.87)	5.01 (1.52, 16.55)
PBC score	Higher PBC score	1.0	1.0	1.0	1.0
Lower PBC score	3.43 (1.06, 11.11)	1.33 (0.16, 11.33)	2.00 (1.00, 4.03)	0.66 (0.26, 1.65)
Social norm score	Higher social norm	1.0	1.0	1.0	1.0
Lower social norm	10.48 (1.88, 58.40)	2.29 (0.13, 41.25)	3.00 (1.40, 6.42)	0.84 (0.29, 2.38)
Habits	Higher habit strength	1.0	1.0	1.0	1.0
Lower habit strength	10.30 (1.64, 64.62)	1.60 (0.08, 30.65)	4.48 (2.14, 9.36)	1.79 (0.58, 5.52)

PBC: perceived behavioural control; +: adjusted for age and sex only, ‡ adjusted for all other variables included in the model. SA: strongly agree; A: agree; N: neither; SD: strongly disagree; D: disagree. n.s.: not significant; –: not significant in minimally adjusted models; n/a: Models adjusted only for age and sex not presented. Data collected in 2009 and 2010 in Cambridge, UK.
