# Supplementary material for: Patterns and predictors of changes in active commuting over 12 months
Source: Prev Med. 2013 Dec;57(6):776–84. doi: 10.1016/j.ypmed.2013.07.020 (PMC3842498; doi:10.1016/j.ypmed.2013.07.020)
Supplement: Supplementary file 1 — Supplementary material. [file mmc1.doc]

**Appendix A: Further details of classification of the usual mode(s) of travel to and from work**

Using all reported modes of travel over the last seven days, we derived the most frequently reported mode or combination of modes used on the journey and participants were classified into eight summary categories of the most frequently reported mode at each time point comprising: (i) ‘car or motorbike only’, (ii) ‘walking only’, (iii) ‘cycling only’, (iv) ‘bus or train in combination with walking or cycling’, (v) ‘bus or train only’, (vii) ‘car in combination with walking or cycling’ (vii) ‘bus or train and car in combination with walking or cycling’, (viii) ‘bus or train and car’. In those cases where there was a tie for the most frequently reported mode or combination of modes over the seven day period (n=46), we made the conservative assumption of assigning participants to the least active category. For example, (i) when both of the tied modal combinations involved some walking or cycling (e.g. ‘bus and walking’ and ‘car and walking’), we assigned participants to the ‘car and walking’ category; (ii) when the tie was between an ‘active’ and an ‘inactive’ response (e.g. ‘cycling only’ and ‘car only’), we assigned participants to the ‘car only’ category. The summary categories were chosen because they allowed differentiation between those individuals who reported walking or cycling as part of their journeys and those who did not, and were relatively homogenous with respect to socio-demographic characteristics and total weekly time spent walking or cycling for transport.

In order to capture any changes in usual travel mode(s) — in particular towards walking and cycling and alternatives to the car (modal shift) — and due to small numbers of participants making particular shifts (e.g. a shift from ‘car only’ to ‘walking’: n=1) we further classified participants into four broad categories: (i) used alternatives to the car to both time points; (ii) used the car at both time points; (iii) shifted from the car to an alternative and (iv) shifted from an alternative mode to the car (Table A1).

**Table A1:** Detailed breakdown of change in most frequently reported travel mode(s)

|  | |  | **t2** | | | | | | | |  |
| --- | --- | --- | --- | --- | --- | --- | --- | --- | --- | --- | --- |
|  | |  | Car or  Motorbike only | Walking  only | Cycling  only | PT and walking / cycling | PT | Car and active | PT and car and active | PT and car | Total |
| **t1** | | Car or motorbike only | 137 | 1 | 9 | 2 | 1 | 20 | 0 | 4 | 174 |
|  |  | Walking only | 2 | 28 | 7 | 0 | 1 | 0 | 0 | 0 | 38 |
|  |  | Cycling only | 17 | 8 | 246 | 1 | 6 | 4 | 1 | 0 | 283 |
|  |  | PT and walking/cycling | 0 | 1 | 3 | 6 | 6 | 3 | 0 | 1 | 20 |
|  |  | PT | 4 | 1 | 2 | 5 | 30 | 1 | 0 | 1 | 44 |
|  |  | Car and active | 10 | 3 | 6 | 0 | 4 | 45 | 0 | 2 | 70 |
|  |  | PT and car and active | 0 | 0 | 0 | 0 | 0 | 0 | 2 | 0 | 2 |
|  |  | PT and car | 4 | 0 | 1 | 0 | 3 | 5 | 1 | 10 | 24 |
|  | | Total | 174 | 42 | 274 | 14 | 51 | 78 | 4 | 18 | 655 |
|  |  | | | | | | | | | | |
|  | Car user at both time points | | | | | | | | | | |
|  | Switched from car to alternative | | | | | | | | | | |
|  | Switched from alternative to the car | | | | | | | | | | |
|  | Used alternative to the car at both time points | | | | | | | | | | |

**Appendix B: Further details of objectively assessed environmental variables**

Using a geographical information system (ArcGIS, version 9.3), characteristics of the areas surrounding the home, workplace and route were derived using home and work postcodes geocoded using the Ordnance Survey (OS) Address Layer 2®database (Ordnance Survey, 2012). A pedestrian route network dataset was constructed by combining road data from the OS MasterMap® Integrated Transport Network™ (ITN) database with local authority data on rights-of-way (public footpaths, bridleways and byways), information on cycle routes from the charity Sustrans (Sustrans, 2012), and other informal pathways recorded on OpenStreetMap.com.

Only variables which were associated with travel behaviour in cross-sectional analyses were included as potential predictors here. Based on the Census Output Area in which the home postcode was located, urban rural status and area-level deprivation of home location were assigned; participants were classified as living in either an urban area (with a population of >10000) or in a rural location (town or fringe, village, hamlet or isolated dwelling)[[1]](#endnote-2), deprivation was assessed using the index of multiple deprivation (IMD) scores[[2]](#endnote-3), and both were dichotomised at their median values.

The **National Public Transport Access Node** (NaPTAN) database was used to compute all public transport measures and was provided by the Department for Transport.[[3]](#endnote-4) The shortest network distances from home to the nearest railway station and the nearest bus stop were derived. 800m buffers were delineated around each participant’s home and workplace. This distance was chosen as it is hypothesised to represent the areas that could be accessed within an approximate ten-minute walk and has been used previously in other studies.[[4]](#endnote-5) A count of the bus services running in the neighbourhood was derived and two groups were created (less and more frequent). The number of destinations within 800m of work was also derived and two groups created using the median value as the cutpoint. Destinations included leisure facilities, shops and schools which were obtained from Points of Interest [[5]](#endnote-6).

Participants also reported their workplace or the area of Cambridge where it was located. As our sample lived either: (i) in Cambridge itself or (ii) in the surrounding towns, villages and rural areas and worked either (i) in the heart of the city (<2km from the city centre) or (ii) on the outskirts (>2km from the city centre), we summarised the geographical context of their travel to work using one of four categories based on these characteristics of the home and work location. We hypothesised that those car users who commuted into the heart of the city might be less likely to drive all the way (using the car only, rather than a combination of modes) to avoid traffic congestion in the inner city.

**Appendix C:** Detailed breakdown of change in trips and time in those participants whose weekly time spent walking and cycling increased or decreased

|  | Percentage (n) | | | |
| --- | --- | --- | --- | --- |
|  | Walking | | Cycling | |
|  | Decrease | Increase | Decrease | Increase |
| Same number of trips and increased duration per trip | 0 (0) | 7.6 (10) | 0 (0) | 19.2 (27) |
| Same number of trips and decreased duration per trip | 8.3 (9) | 0 (0) | 14.5 (24) | 0 (0) |
| Increased number of trips and same duration per trip | 0 (0) | 23.7 (31) | 0 (0) | 36.9 (52) |
| Increased number of trips and increased duration per trip | 0 (0) | 49.6 (65) | 0 (0) | 19.2 (27) |
| Increased number of trips and decreased duration per trip | 5.5 (6) | 15.3 (0) | 8.4 (14) | 18.4 (26) |
| Decreased number of trips and same duration per trip | 30.3 (33) | 0 (0) | 34.3 (57) | 0 (0) |
| Decreased number of trips and increased duration per trip | 22.0 (24) | 3.8 (5) | 16.3 (27) | 6.4 (9) |
| Decreased number of trips and decreased duration per trip | 33.0 (36) | 0 (0) | 26.5 (44) | 0 (0) |

Column percentages are given

**References for Appendices:**

Bibby and Shepherd, Developing a New Classification of Urban and Rural

Areas for Policy Purposes – the Methodology, ONS 2004. Available from the ONS website: <http://www.statistics.gov.uk/geography/downloads/Methodology_Report.pdf> Last accessed: 22.07.12

^2^ Department for Communities and Local Government 2011. English Indices of Deprivation 2010.

^3^ Department for Transport, NaPTAN, 2012. Available from: <http://www.dft.gov.uk/naptan/>. Last accessed: 22.07.12

^4^ Van Dyck D, Deforche B, Cardon G, De Bourdeaudhuij I., 2009. Neighbourhood walkability and its particular importance for adults with a preference for passive transport. Health & Place; 15: 496-504.

^5^ PointX Ltd, 2010. Ordnance Survey Points of Interest. Southampton: Ordnance Survey. Available at www.ordnancesurvey.co.uk/oswebsite/products/points-of-interest/index.html. Last accessed 20.09.13.

1. Bi and Shepherd, (2004) Developing a New Classification of Urban and Rural

   Areas for Policy Purposes – the Methodology Available from the ONS website:

   [htp://www.statistics.gov.uk/geography/downloads/Methodology_Report.pdf](http://www.statistics.gov.uk/geography/downloads/Methodology_Report.pdf) Last accessed: 22.11.12 [↑](#endnote-ref-2)
2. De [↑](#endnote-ref-3)
3. Available from: <http://www.dft.gov.uk/naptan/>. Last accessed: 22.11.12 [↑](#endnote-ref-4)
4. forche B, Cardon G, De Bourdeaudhuij I (2009) Neighbourhood walkability and its particular importance for adults with a preference for passive transport. *Health & Place* 15: 496-504. [↑](#endnote-ref-5)
5. [↑](#endnote-ref-6)
